# Restoration of Cementoblast Mineralization by Glutamine Under Inflammatory Conditions

**DOI:** 10.1016/j.identj.2025.103930

**Published:** 2025-10-07

**Authors:** Mengjie Yin, Hantao Yao, Jingqiu Chen, Ting Li, Yilin Liao, Yaoyu Zhao, Yue Sheng, Wengwanyue Ye, Zhengguo Cao, Yaoting Ji, Hong He

**Affiliations:** a State Key Laboratory of Oral & Maxillofacial Reconstruction and Regeneration, Key Laboratory of Oral Biomedicine Ministry of Education, Hubei Key Laboratory of Stomatology, School & Hospital of Stomatology, Wuhan University, Wuhan, China; b Stomatology Hospital, School of Stomatology, Zhejiang University School of Medicine, Zhejiang Provincial Clinical Research Center for Oral Diseases, Zhejiang Key Laboratory of Oral Biomedical, Cancer Center of Zhejiang University, Engineering Research Center of Oral Biomaterials and Devices of Zhejiang Province, Hangzhou 310000, China; c Department of Periodontology, School & Hospital of Stomatology, Wuhan University, Wuhan, China; d Department of Orthodontics, School & Hospital of Stomatology, Wuhan University, Wuhan, China

**Keywords:** Glutamine, GLS, *P. gingivalis*, Cementoblast, Cell mineralization, Dental cementum

## Abstract

**Introduction and Aims:**

Cementoblasts are critical for cementum formation and periodontal regeneration during inflammatory diseases such as periodontitis and apical periodontitis (AP). Bacterial infection leads to metabolic changes in cementoblasts and reduces mineralization capability. This study aimed to fully elucidate the metabolic changes of cementoblasts in inflammatory mineralization and explore therapeutic strategies targeting metabolism.

**Methods:**

Mouse cementoblasts cell line OCCM-30 were infected with *Porphyromonas gingivalis* (*P.g*) to model microbial infection stress. Metabolic profiling was performed using Seahorse assays and untargeted metabolomics. Genetic manipulation (knockdown/overexpression of *Gls*) and pharmacological inhibition (AMPK inhibitor Compound C) were used to dissect the mechanisms. An AP model was induced in C57BL/6 mice, and glutamine supplementation was administered to assess therapeutic effects *in vivo*.

**Results:**

*P.g* infection suppressed glycolysis and oxidative phosphorylation but upregulated glutamine metabolism in cementoblasts. Knockdown of *Gls* inhibited mineralization by the cementoblasts, whereas glutamine supplementation or *Gls* overexpression restored mineralization ability. AMPK inhibition reduced GLS expression and mineralization by cementoblasts. *In vivo*, glutamine supplementation attenuated AP progression and cementum destruction.

**Conclusion:**

Glutamine supplementation compensates for energy deficits in inflamed cementoblasts via the AMPK/GLS signalling pathway. Targeting this pathway offers a potential therapeutic approach for cementum remineralization and periodontal regeneration.

**Clinical Relevance:**

Glutamine supplementation restores mineralization by the cementoblasts under inflammatory conditions, which provides a novel theoretical foundation for the alleviation and treatment of periodontitis and apical periodontitis.

## Introduction

Periodontitis and apical periodontitis are chronic inflammatory diseases that progressively destroy the cementum, periodontal ligament and alveolar bone, leading to tooth loss.^1^^,^^2^ *Porphyromonas gingivalis* (*P.g*) is a common pathogen in apical periodontitis.^3^^,^^4^ Cementum is a layer of mineralised tissue covering the tooth root with vital function in tooth attachment and force transmission. Its regeneration relies on the functional activity of cementoblasts, specialised cells responsible for cementum formation and repair,^5^, ^6^, ^7^ which has always been valued as the gold standard of periodontal tissue regeneration.^8^^,^^9^ Cementoblasts show a mineralization process similar to osteoblasts and express related mineralization markers.^10^, ^11^, ^12^ While significant progress has been made in understanding the molecular pathways governing cementoblast differentiation,^13^, ^14^, ^15^, ^16^, ^17^ the impact of microbial flora which significantly affects the metabolic microenvironment has unique research value in cementoblasts. Emerging evidence highlights the interplay between cellular metabolism and bone homeostasis.^18^^,^^19^ Glycolysis and oxidative phosphorylation have proven to be critical for osteoblast mineralization,^20^ whereas metabolic shifts under inflammatory conditions can impair bone formation.^21^^,^^22^ Glycolysis has been demonstrated to be associated with macrophage senescence, thereby influencing the progression of periodontitis.^23^ As a keystone pathogen in periodontitis, *P.g* disrupts host–microbe equilibrium and induces mitochondrial dysfunction in periodontal cells.^24^^,^^25^ However, how *P.g* reprograms cementoblast metabolism to compromise mineralization remains unknown.

Glutamine (Gln), the most abundant non-essential amino acid, plays an important role in many physiological processes.^26^^,^^27^ It has been established that exogenous glutamine is essential for the survival and metastasis of cancer cells.^28^, ^29^, ^30^, ^31^ Glutamine metabolism is also significantly enhanced in bone remodelling by promoting the proliferation of skeletal stem cells and restored mechanosensitivity of osteoblast.^32^^,^^33^ Glutaminase (GLS) is the key enzyme initiating glutamine metabolism, catalysing the hydrolysis of glutamine into glutamate and ammonium ions.^34^ The inhibition of GLS has been demonstrated to inhibit the progression of different kinds of cancers.^30^^,^^35^ The decomposition product of glutamine is necessary for the proliferation of skeletal stem cells^32^; however, the role of glutamine metabolism mediated by GLS in cementoblasts has not been elaborated yet.

Insufficient energy generation will cause energy stress, which will activate alternative metabolic pathways to compensate for the energy needs. AMP-activated protein kinase (AMPK), a central energy sensor, is activated during energy stress to promote catabolic pathways.^36^ Studies have shown that the AMPK-Skp2-Akt axis regulates EGF-induced glucose metabolism and cancer cell migration,^37^ whereas recent work links AMPK to glutaminolysis via the PDZD8/GLS1 axis in cancer,^38^ but whether this axis operates in cementoblasts to counteract *P.g*-induced energy deficits is unclear.

In this study, we investigated metabolic alterations in cementoblasts under *P.g* infection using a combination of multi-omics approaches, genetic manipulation and mouse models. We demonstrated that *P.g* suppresses both glycolysis and oxidative phosphorylation while concurrently activating AMPK/GLS-mediated glutaminolysis to alleviate energy stress. These findings uncover a previously unrecognised metabolic adaptation and suggest that targeting glutamine metabolism may represent a promising therapeutic strategy for periodontal regeneration.

## Materials and methods

### Bacterial culture

*P. gingivalis* (P.g) strain W83 was provided by the School of Stomatology, Wuhan University (China), and cultured in 30 g/L trypticase soy broth (TSB) in an anaerobic tank (80% N_2_, 10% CO_2_ and 10% H_2_) at 37 °C. A combination of 5 g/L yeast extract, 0.5 g/L L-cysteine hydrochloride, 5 mg/L hemin and 1 mg/L menadione was added to the culture medium. Bacteria counts were measured by the OD value at 600 nm, and the number of bacteria was 10^9^/mL at OD = 1.^14^

### Cell culture and treatment

The immortalised mouse cementoblast cell line OCCM-30 was kindly provided by Dr. Martha J. Somerman of the National Institutes of Health, Bethesda, MD, USA.^39^ The cells were cultured in high-glucose Dulbecco's Modified Eagle medium (DMEM) containing 10% fetal bovine serum (FBS) in a humidified atmosphere at 37 °C with 5% CO_2._^39^ Cementogenic medium (CM) containing DMEM, 5% FBS, 10 mM β-glycerophosphate disodium, 50 μg/mL L-ascorbic acid and 10 nM dexamethasone was used to induce mineralization by the cementoblasts when the cell density reached 80%. Exogenous tumour necrosis factor α (TNF-α) and Gln were added to the CM as the experimental design.

### RNA isolation and quantitative real-time polymerase chain reaction (qPCR) analysis

Total RNA was extracted using Trizol reagents. The concentration of total RNA was determined by NanoDrop 2000/2000c spectrophotometer at 260 nm and 280 nm, and 1 μg RNA was subsequently used for reverse transcription for cDNA synthesis using PrimeScriptTM Reverse Transcription Reagent Kit (Vazyme, China). Applied Biosystems QuantStudio 6 was employed to conduct qPCR using SYBR qPCR Master Mix. The expression levels of genes were calculated by 2^-ΔΔCt^ method and normalised by the internal reference gene Rn18s (18s). The detailed sequences of specific primers are provided in Table 1.

**Table 1:** Primer sequences used for q-PCR.

Genes	Forward	Reverse
*18s*	AGTCCCTGCCCTTTGTACACA	CGATCCGAGGGCCTCACTA
*Runx2*	GGGAACCAAGAAGGCACAGA	GGATGAGGAATGCGCCCTAA
*Sp7*	AGTGGGAACAAGAGTGAGCTG	TAGTGAGCTTCTTCCTGGGT
*Col1a1*	CGATGGATTCCCGTTCGAGT	CGATCTCGTTGGATCCCTGG
*Ocn*	TTCTGCTCACTCTGCTGACC	GGGACTGAGGCTCCAAGGTA
*Alp*	GCACCTGCCTTACCAACTCT	GTGGAGACGCCCATACCATC
*Gls*	GTCTGGAGGGAAGGTTGCTG	TGCCTTTGCCCATCTACAGT
*Il-1β*	AATGCCACCTTTTGACAGTGATG	AGCTTCTCCACAGCCACAAT

### Protein extraction and Western blot

Cell protein was extracted using RIPA lysis buffer with phosphatase inhibitor and protease inhibitor added. Protein quantification was performed using a BCA protein assay kit, and equal amounts of protein in each sample (30 μg) were subsequently separated by 10% sodium dodecyl sulfate—polyacrylamide gel electrophoresis (SDS-PAGE) at 230 V for 35 min, after which the protein was transferred onto a 0.45-μm PVDF membrane pretreated with absolute ethyl alcohol at 400 mA for 40 min. The membranes were blocked in a protein-free fast-blocking solution for 5 min and incubated with primary antibodies at 4 °C overnight. After 3 washes of TBST, the membranes were incubated with HRP-conjugated secondary antibodies at room temperature for 1 h and the protein signals of interest were detected using Enhanced Chemi Luminescence. The details of specific antibodies are provided in Table 2.

**Table 2:** Antibodies used for Western blot.

Antibodies	Working concentration	Source
Anti-RUNX2	1:500	Boster, China
Anti-SP7	1:1,000	Abcam, USA
Anti-BSP	1:500	Affinity Biosciences, USA
Anti-OCN	1:100	Santa Cruz Biotechnology, USA
Anti-ALP	1:1,000	ABclonal, China
Anti-GLS	1:1,000	Proteintech, China
Anti-AMPK-α	1:1,000	Cell Signaling Technology, USA
Anti-p-AMPK-α	1:1,000	Cell Signaling Technology, USA
Anti-mTOR	1:800	ABclonal, China
Anti-S6	1:1,000	Cell Signaling Technology, USA
Anti-p-S6	1:800	Cell Signaling Technology, USA
Anti-β-actin	1:5,000	Proteintech, China

### Alkaline phosphatase (ALP) staining and ALP activity assay

After 4 days of cementogenic induction, OCCM-30 cells were washed with phosphate-buffered saline (PBS) and fixed with 4% paraformaldehyde for 15 min, after which the cells were stained using the BCIP/NBT Alkaline Phosphatase Color Development Kit. After 4 days of cementogenic induction, OCCM-30 cells were lysed with RIPA lysis buffer. After centrifugation at 12,000 × g at 4 °C for 15 min, the supernatant was collected to perform an ALP activity assay using an ALP assay kit. Protein quantification was simultaneously performed using a BCA protein assay kit to normalize ALP activity data.

### Seahorse analysis

The oxygen consumption rate (OCR) was measured using a Seahorse XF Cell Mito Stress Test Kit, and the proton efflux rate (PER) was measured using a Seahorse XF Glycolytic Rate Assay Kit. Briefly, OCCM-30 cells were digested and seeded into XFe96 microplates at a density of 2 × 10^4^ cells/well after 4 days of cementogenic induction. The detection system was preheated the day before the experiment, and XF calibrant was added to a utility plate covered with a sensor cartridge to hydrate the probe in 37 °C CO_2_-free incubator overnight. The next day, the test solution and the compounds were prepared according to the protocol. The working concentrations of each compound in the Seahorse XF Cell Mito Stress Test were 1.5 μM for oligomycin (Oligo), 2.0 μM for carbonyl cyanide 4-(trifluoromethoxy) phenylhydrazone (FCCP) and 0.5 μM for the mixture of rotenone and antimycin A (Rot/AA). The working concentrations of each compound in the Seahorse XF Glycolytic Rate Assay were 0.5 μM for Rot/AA and 50 mM for 2-Deoxy-D-glucose (2-DG). After cells were washed, the sensor cartridge and the XFe96 microplate were loaded into a Seahorse XFe96 Analyzer for testing. Data were analysed using Agilent's Seahorse Wave Controller.

### RNA sequencing and analysis

After 4 days of cementogenic induction with or without *P.g*, total RNA of OCCM-30 cells was isolated using the Trizol Reagent, and the purity and integrity of the total RNA was subsequently qualified. Sequencing libraries were generated using Hieff NGS® mRNA Isolation Master Kit and sequenced on DNBSEQ-T7 with PE150 model at Bioyi Biotechnology. StringTie (v2.1.5) statistics was used to compare the Read Count values on each gene as the original expression of the gene and then use FPKM to standardize the expression. The following analysis was conducted using a free online platform (www.bioinformatics.com.cn).

### Untargeted metabolomics sequencing

After 4 days of cementogenic induction with or without *P.g*, OCCM-30 cells were digested and washed with PBS. After centrifugation at 1,000 × g at 4 °C for 5 min, the supernatant was discarded, and the precipitated cells were quick-frozen in liquid nitrogen and stored at –80 °C. The samples were sent for untargeted metabolomics performed by Liquid Chromatography–Mass Spectrometry (LC-MS). LC-MS/MS analysis was performed using an Ultra High Performance Liquid Chromatography (UHPLC) system. The raw data were converted to the mzXML format using ProteoWizard and processed with an in-house program which was developed using R and based on XCMS for peak detection, extraction, alignment, and integration. The heatmap analysis was conducted using a free online platform (www.bioinformatics.com.cn).

### Lentiviral packaging and cell infections

Plasmids including packaging plasmids pMD2.G and psPAX2 along with the plasmids pLV2-U6-Ctrl, pLV2-U6-Gls, pLV3-CMV-Ctrl and pLV3-CMV-Gls were constructed by Wuhan Miaoling Biotechnology. Invitrogen’s Lipofectamine 2000 reagent was used to transfect the plasmids into HEK293T cells. After 48 h of transfection, the supernatant containing different lentiviruses was collected and filtered by 0.45-μm filters. For cell infections, OCCM-30 cells were seeded on 24-well plates and a mixture of 50% DMEM and 50% lentivirus supernatant was used to infect OCCM-30 cells at a cell density of 30%. Polybrene (5 μg/mL) was added to the mixture to enhance infection efficiency, and after a 12-h incubation, 5 μg/mL puromycin was used to select successfully infected cells.

### Animal experiment

Animal experiments in this study were approved by the Ethics Committee of the School of Stomatology at Wuhan University (approval number S07924010D). A total of 18 7-week-old specific pathogen-free C57BL/6 male mice were purchased from the Experimental Animal Center of Wuhan and housed under light-, temperature-, and humidity-controlled conditions (12-h light/dark cycle; 22 ± 2 °C; 55 ± 5% humidity) with free access to food and water. After 1 week of adaptive feeding, they were randomly allocated into 3 groups (6 per group): the healthy group, the AP group and the AP plus 0.5% Gln group. The first molars on both sides of the mandible were drilled with a 1/4 round bur to establish pulp exposure,^40^^,^^41^ and the healthy group underwent a sham surgery. From the next day on, 0.5% glutamine was added to the drinking water in the experimental group, with the water refilled every other day. The healthy group was given regular sterile water. On the 21st day, all mice were euthanised, and the mandibles were collected and fixed in 4% paraformaldehyde solution.

### Micro-computed tomography (micro-CT) and histological analysis

After being fixed in a 4% paraformaldehyde solution for 48 h, the mandibles were subsequently scanned using the Skyscan 1176 micro-CT instrument. The scanning parameters were set as 70 kV, 200 μA, with a resolution of 6 μm. The CTAn software was used to view the reconstructed sagittal and coronal images as well as to quantitatively calculate the volume of apical lesions (*n* = 10). After micro-CT scanning, the mandibles were decalcified in 10% ethylenediaminetetraacetic acid (EDTA) for 4 weeks, and after alcohol gradient dehydration and paraffin embedding, 4-μm slices were sectioned for histological analysis. The sections were dewaxed and dehydrated for haematoxylin and eosin (H&E), immunofluorescence (IF) and immunohistochemical (IHC) staining (*n* = 3). The slices for IF staining were incubated with the primary antibody RUNX2 (1:100) at 4 °C overnight and treated with fluorescent-labeled secondary antibody for 1 h at room temperature followed by the application of antifade mounting medium with 4′,6-diamidino-2-phenylindole (DAPI) the next day. The IHC staining was conducted using an UltraSensitive^TM^ SP kit while the slices were incubated with primary antibody ALP (1:100). Finally, the slices were stained with 3,3′-diaminobenzidine (DAB) for 1 min and haematoxylin staining was used for the cell nuclei coloration.

### Statistical analysis

All experiments were performed in at least 3 independent replicates. The experimenters responsible for the outcome assessment and data analysis were unaware of the group allocation. Data were analysed using GraphPad Prism 9.0, and the results were presented as mean ± standard error of the mean (SEM). Comparisons between 2 groups were evaluated by unpaired Student's *t*-test while data among multiple groups were compared by one-way analysis of variance (ANOVA). *P* < 0.05 was considered a significant statistical difference.

## Results

### P.g inflammation inhibits mineralization by cementoblasts

**Fig. 1:**
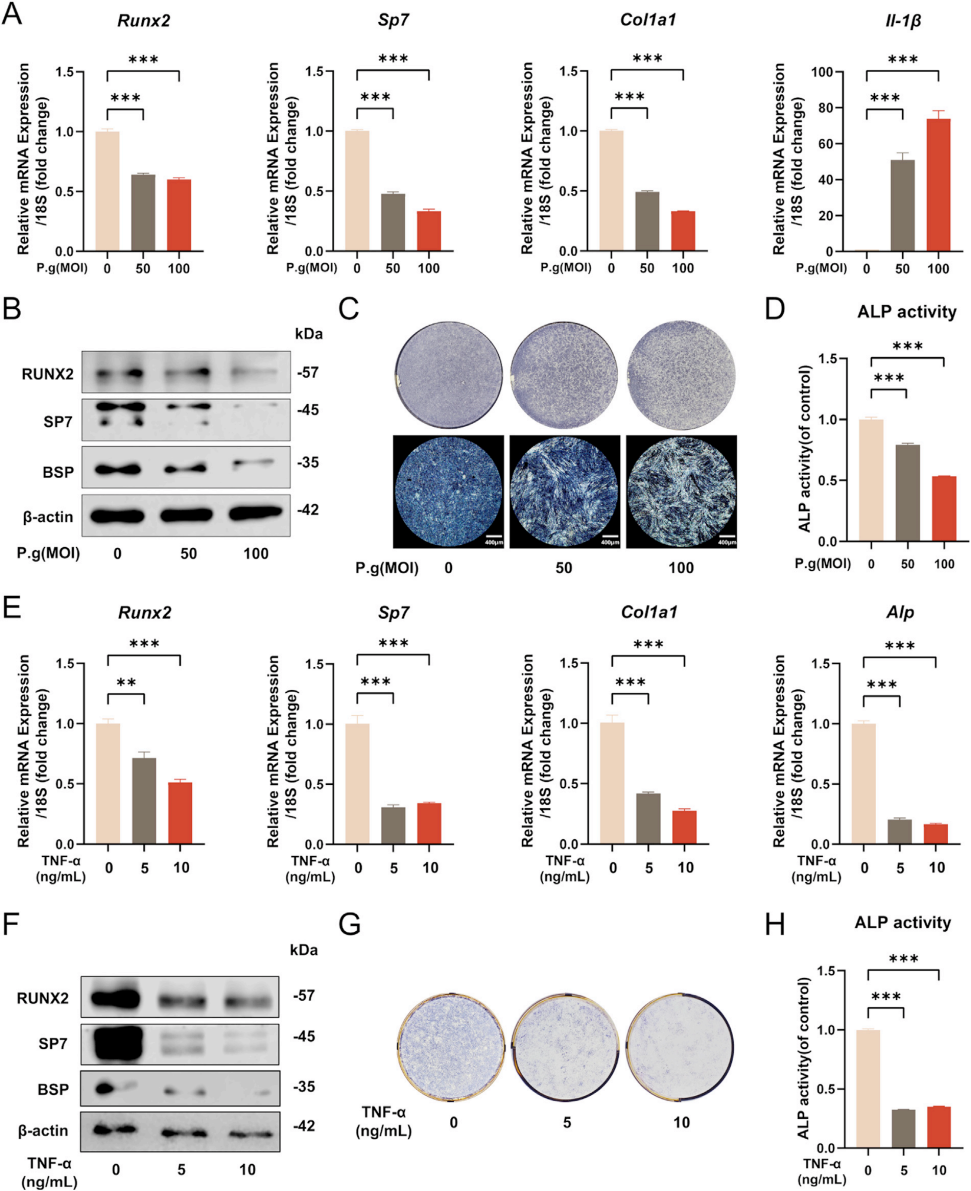
*P.g* inhibits mineralization by cementoblast. A-B, OCCM-30 cells were mineralization induced with *P.g* at different MOIs (MOI = 0, 50, and 100) for 4 days. *Runx2, Sp7, Col1a1* and *IL-1β* expression levels were detected by qPCR (A), and RUNX2, SP7 and BSP expression levels were detected by Western blot (B). C-D, ALP staining (C) and ALP activity assay (D) conducted with *P.g* treatment at different MOIs (MOI = 0, 50, and 100) for 4 days of mineralization induction. E-F, OCCM-30 cells were mineralization induced with TNF-α at different concentrations (0, 5, and 10 ng/mL) for 4 days. *Runx2, Sp7, Col1a1* and *Alp* expression levels were detected by qPCR (E) and RUNX2, SP7 and BSP expression levels were detected by Western blot (F). G-H, ALP staining (G) and ALP activity assay (H) conducted with TNF-α at different concentrations (0, 5, and 10 ng/mL) for 4 days of mineralization induction. Data are presented as mean ± SEM. *P < .05, ^⁎⁎^*P* < .01, ^⁎⁎⁎^*P* < .001. Biological replicates for each experiment, *n* = 3.

To detect the effect of *P.g* on mineralization by the cementoblasts, we treated mouse cementoblast cell line OCCM-30 with the main periodontal pathogen *P.g* in different multiplicity of infections (MOI = 0, 50, 100) and performed mineralization induction for 4 days. As shown in Figure 1A, the mRNA expression levels of mineralization markers *Runx2, Sp7* and *Col1a1* significantly decreased while the inflammatory factor *IL-1β* increased dramatically. Consistently, the protein expression of RUNX2, SP7 and BSP gradually decreased with *P.g* treatment (Figure 1B). To determine the alteration of mineralization ability, we performed ALP staining, which also showed an inhibitory effect of *P.g* on cementoblasts (Figure 1C). Similarly, ALP activity also decreased as indicated with colorimetry (Figure 1D). To verify that the inhibitory effect of *P.g* on mineralization was caused by its pro-inflammatory effect, we simultaneously established a TNF-α inflammation model by adding exogenous TNF-α to the mineralization induction medium. After 4 days of mineralization induction, the mRNA level of *Runx2, Sp7, Col1a1* and *Alp* significantly decreased with exogenous TNF-α treatment (Figure 1E). Additionally, the protein level of RUNX2, SP7 and BSP gradually decreased 4 days after TNF-α treatment (Figure 1F). ALP staining and ALP activity assay also consistently decreased after TNF-α treatment (Figure 1G and H). The above results suggest that the inflammatory environment caused by *P.g* infection can suppress mineralization by cementoblasts.

### Energy metabolism was inhibited during inflammatory mineralization by cementoblasts

**Fig. 2:**
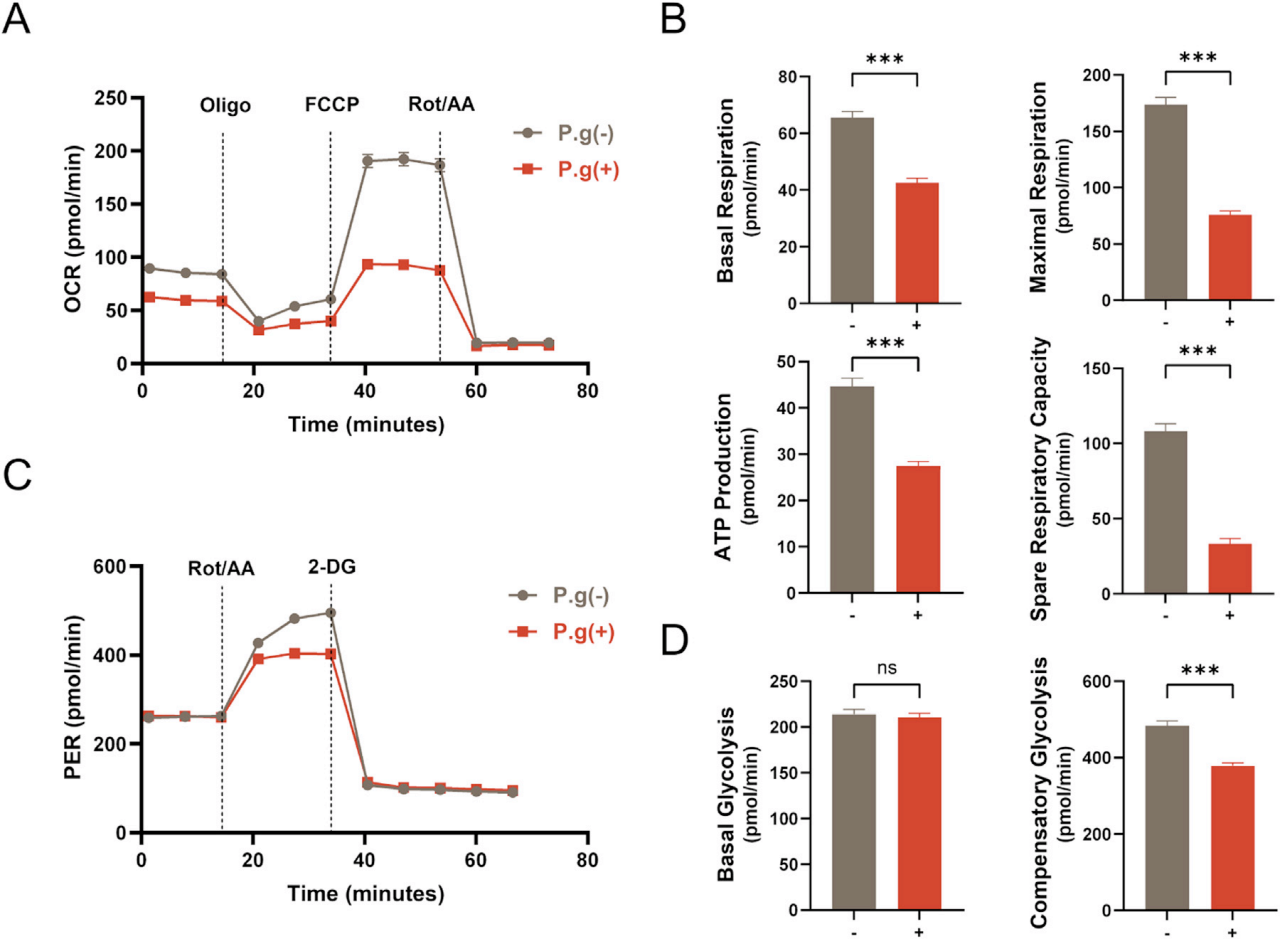
Energy metabolism was inhibited during inflammatory mineralization by cementoblasts. A, OCR data from a mitochondrial stress test of OCCM-30 cells treated with or without *P.g*. Oligo, oligomycin; FCCP, carbonyl cyanide 4-(trifluoromethoxy)phenylhydrazone; Rot/AA, rotenone + antimycin A. B, Quantification of the indicated OCR parameters. C, PER data from a glycolysis stress test of OCCM-30 cells treated with or without *P.g*. 2-DG, 2-deoxyglucose. D, Quantification of the indicated PER parameters. Data are presented as mean ± SEM. *P < .05, ^⁎⁎^*P* < .01, ^⁎⁎⁎^*P* < .001 and *ns* for not significant. Biological replicates for each experiment, *n* = 6.

The energy metabolic alternations of cementoblast under *P.g* infection were investigated using Seahorse analysis. The oxygen consumption rate (OCR) decreased remarkably in cementoblasts treated with *P.g* (Figure 2A). Furthermore, the quantification of OCR parameters such as basal respiration, maximal respiration and spare respiratory capacity were all depressed by *P.g*, with a lower level of ATP production (Figure 2B). The extracellular acidification rate (ECAR) was transformed into a rate that reflects the number of protons extruded over time, the proton efflux rate (PER). Similarly to OCR, the PER was lower in cementoblasts treated with *P.g* (Figure 2C), and so were the quantification parameters of compensatory glycolysis, whereas the basal glycolysis had no significant change (Figure 2D). These findings suggest that both glycolysis and oxidative phosphorylation are significantly inhibited during the inflammatory mineralization process of cementoblasts.

### Multi-omics revealed that glutamine metabolism plays an important role in mineralization by cementoblasts under P.g inflammation

**Fig. 3:**
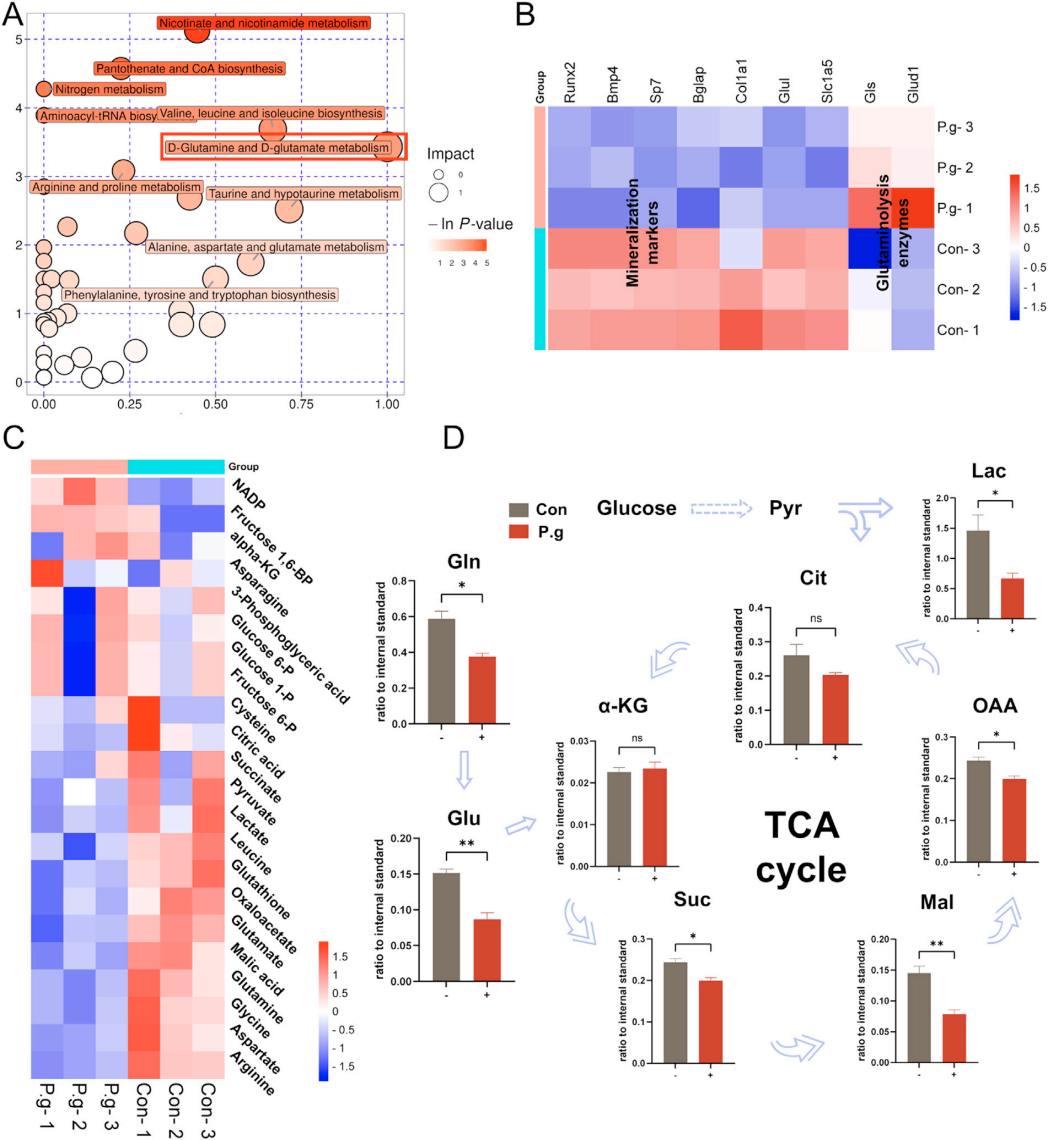
Multi-omics revealed that glutamine metabolism plays an important role in mineralization by cementoblasts under *P.g* inflammation. A, Pathway analysis derived from untargeted metabolomics data illustrating pathways enriched in OCCM-30 cells treated with *P.g* in comparison with those treated without *P.g*. B, A heatmap represents the genes of osteogenic markers and key enzymes involved in glutamine metabolism in OCCM-30 cells treated with or without *P.g*. C, A heatmap represents the metabolites involved in glutamine and glucose metabolism in OCCM-30 cells treated with or without *P.g*. D, A sketch map showing the key metabolite changes in glutaminolysis and the TCA cycle. Data are presented as mean ± SEM. *P < .05, ^⁎⁎^*P* < .01, ^⁎⁎⁎^*P* < .001 and *ns* for not significant. Biological replicates for each experiment, *n* = 3.

To probe these specific alterations, we performed untargeted metabolomics sequencing. Pathway analysis derived from untargeted metabolomics found that glutamine and glutamate metabolism had the greatest impact (Figure 3A). As shown in Figure 3B, the RNA-seq data showed that the key enzymes related to glutamine metabolism were upregulated under *P.g* inflammation. Notably, the expression of gene hallmarks associated with glutamine absorption decreased while those associated with glutaminolysis were significantly upregulated. The heatmap (Figure 3C) showed a significant decrease in some key metabolites in glutamine metabolism and the TCA cycle, from which we drew a schematic representation (Figure 3D). The untargeted metabolomics revealed that the levels of lactate in *P.g*-infected cementoblasts were reduced, consistent with the Seahorse assay results showing a decrease in PER (Figure 2C), indicating inhibited glycolysis. Additionally, the levels of glutamine and glutamate significantly decreased while α-KG levels had no significant change (Figure 3D). Key intermediates levels in the TCA cycle including succinate, malate, oxaloacetate and citrate showed significant reductions, with no statistical difference observed in citrate levels (Figure 3D). The above results suggested that significant metabolic alterations including glutamine metabolism appeared within cementoblasts under the infection of *P.g*.

### Glutamine metabolism mediated by GLS is essential for mineralization by cementoblasts

**Fig. 4:**
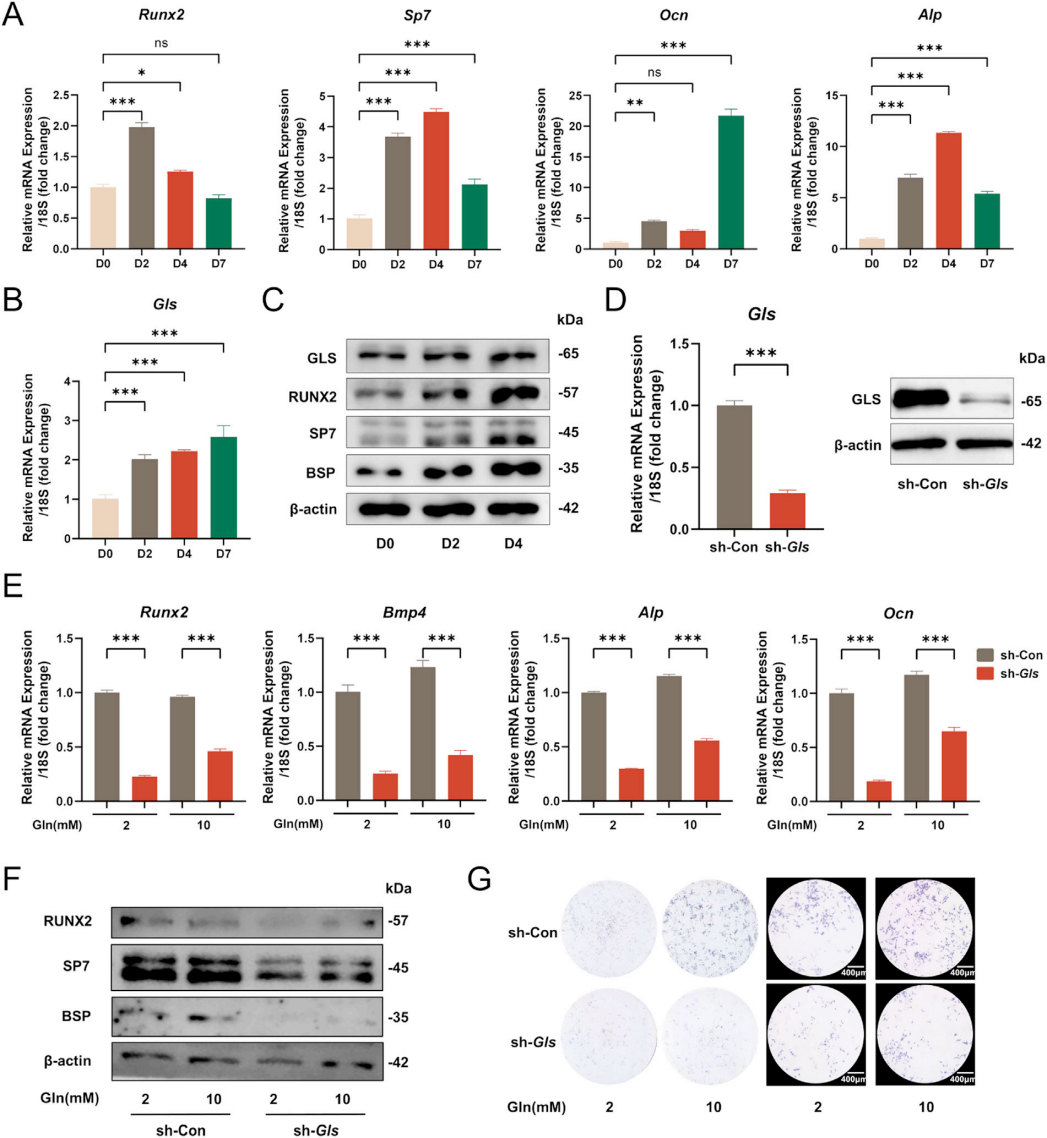
Glutamine metabolism mediated by GLS is essential for mineralization by cementoblasts. A, OCCM-30 cells were mineralization induced for 0, 2, 4, and 7 days. *Runx2, Sp7, Ocn* and *Alp* expression levels were detected by qPCR. B, OCCM-30 cells were mineralization induced for 0, 2, 4, and 7 days. *Gls* expression level was detected by qPCR. C, RUNX2, SP7, BSP and GLS expression levels were detected by Western blot. D, The knockdown efficiency of *Gls* in OCCM-30 cells was tested by qPCR and Western blot. E, *Runx2, Bmp4, Alp* and *Ocn* expression levels in the control (sh-Con) and the *Gls*-knockdown (sh-*Gls*) cells treated with different concentrations of glutamine (2 and 10 mM) after 2 days of mineralization induction were detected by qPCR. F, RUNX2, SP7 and BSP expression levels in the sh-Con and sh-*Gls* cells treated with different concentrations of glutamine (2 and 10 mM) after 2 days of mineralization induction were detected by Western blot. G, ALP staining conducted after 2 days of mineralization induction under different glutamine concentrations (2 and 10 mM) in the sh-Con and sh-*Gls* cells. Data are presented as mean ± SEM. *P < .05, ^⁎⁎^*P* < .01, ^⁎⁎⁎^*P* < .001 and *ns* for not significant. Biological replicates for each experiment, *n* = 3.

To better understand the role of glutamine metabolism in mineralization by cementoblasts, we performed mineralization induction on cementoblasts for 0, 2, 4 and 7 days and observed the change in GLS. The mRNA expression level of *Runx2, Sp7, Ocn* and *Alp* increased significantly on day 2 (Figure 4A), and the mRNA expression level of *Gls* also increased with the extension of the mineralization induction time (Figure 4B). Consistently, the protein levels of RUNX2, SP7, BSP and GLS all increased after 2 and 4 days of mineralization induction (Figure 4C). We next used the lentiviral vector system to construct a stable *Gls-*knockdown cell line in cementoblast. qPCR and Western blot were performed to assess the efficiency of infection (Figure 4D). qPCR showed that the mRNA expression level of *Runx2, Bmp4, Alp* and *Ocn* in cementoblast decreased significantly after knockdown of *Gls* under different concentrations of glutamine (Gln = 2 and 10 mM) (Figure 4E). Protein levels of RUNX2, SP7 and BSP had the same downtrend in the sh-*Gls* group when treated with different concentrations of glutamine (Figure 4F). ALP staining showed that the ability of mineralization was significantly suppressed after knockdown of *Gls* (Figure 4G). These findings are in accordance with the previous study in skeletal stem cells,^32^ thus we believed that glutamine metabolism was upregulated in cementoblast under *P.g* infection, and glutamine metabolism mediated by GLS is essential for mineralization by cementoblasts. Overall, the above findings suggest that glutamine metabolism may be a key metabolic pathway in mineralization by cementoblasts.

### Glutamine supplementary partially restores cementoblast mineralization ability under P.g inflammation

**Fig. 5:**
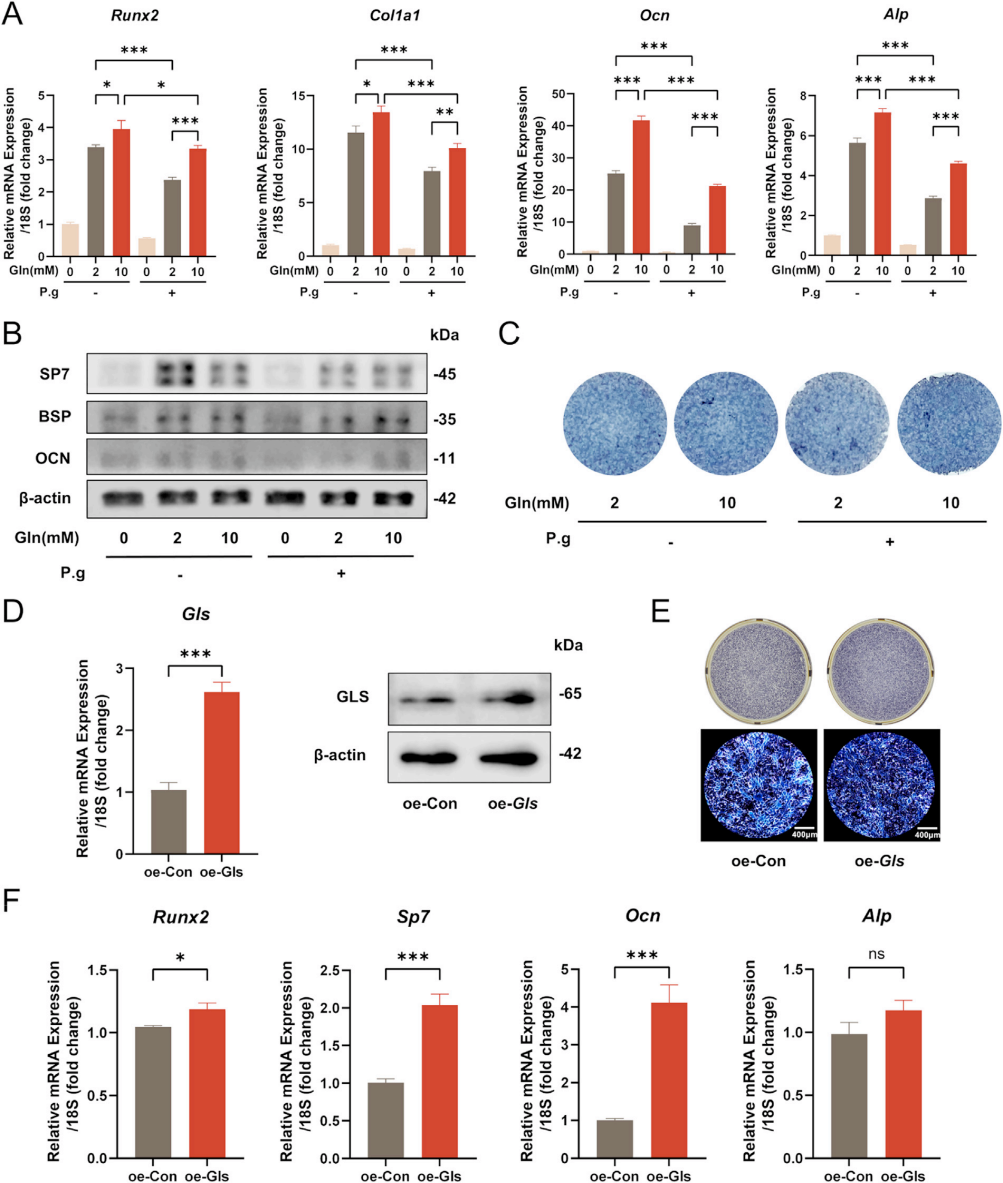
Upregulation of glutamine metabolism partially restores mineralization ability under *P.g* inflammation. A-B, OCCM-30 cells were mineralization induced under different glutamine concentrations (Gln = 0, 2, and 10 mM) with or without *P.g. Runx2, Col1a1, Ocn* and *Alp* expression levels were detected by qPCR (A). SP7, BSP and OCN expression levels were detected by Western blot (B). C, ALP staining was conducted to verify the ability of mineralization. D, The overexpression efficiency of *Gls* in OCCM-30 cells was tested by qPCR and Western blot. E, Control (oe-Con) and Gls-overexpressing (oe-Gls) cells were induced to mineralise with P.g for 4 days, and ALP staining was performed to assess their mineralization ability. F, *Runx2, Sp7, Ocn* and *Alp* expression levels were detected by qPCR. Data are presented as mean ± SEM. *P < .05, ^⁎⁎^*P* < .01, ^⁎⁎⁎^*P* < .001 and *ns* for not significant. Biological replicates for each experiment, *n* = 3.

Considering the importance of GLS-mediated glutamine metabolism in mineralization, we performed a rescue assay to evaluate the effect of glutamine metabolism upregulation on inflammatory mineralization by glutamine supplementation and overexpression of *Gls*. We added glutamine (Gln = 0, 2, 10mM) to the mineralization induction medium for 2 days, and qPCR showed that *Runx2, Col1a1, Ocn* and *Alp* also increased under *P.g* infection by glutamine supplementation (Figure 5A). Western blot results showed that SP7, BSP and OCN were also up-regulated after glutamine supplementation in *P.g* infection (Figure 5B). ALP staining revealed that the mineralization ability was enhanced by glutamine supplementation (Figure 5C). Meanwhile we overexpressed *Gls* in cementoblasts to enhance glutamine metabolism with a lentiviral vector system (Figure 5D). The mineralization ability of the *Gls* overexpression group was significantly increased under *P.g* infection as indicated by ALP staining (Figure 5E). Consistently, the mRNA level of *Runx2, Sp7, Ocn* and *Alp* was up-regulated in the *Gls* overexpression group under *P.g* infection as indicated by qPCR (Figure 5F). The above results suggested that up-regulation of glutamine metabolism from 2 different perspectives can partially restore the ability of mineralization in cementoblasts under *P.g* infection.

### Glutamine supplementary restores mineralization by cementoblasts under P.g inflammation through AMPK/GLS pathway

**Fig. 6:**
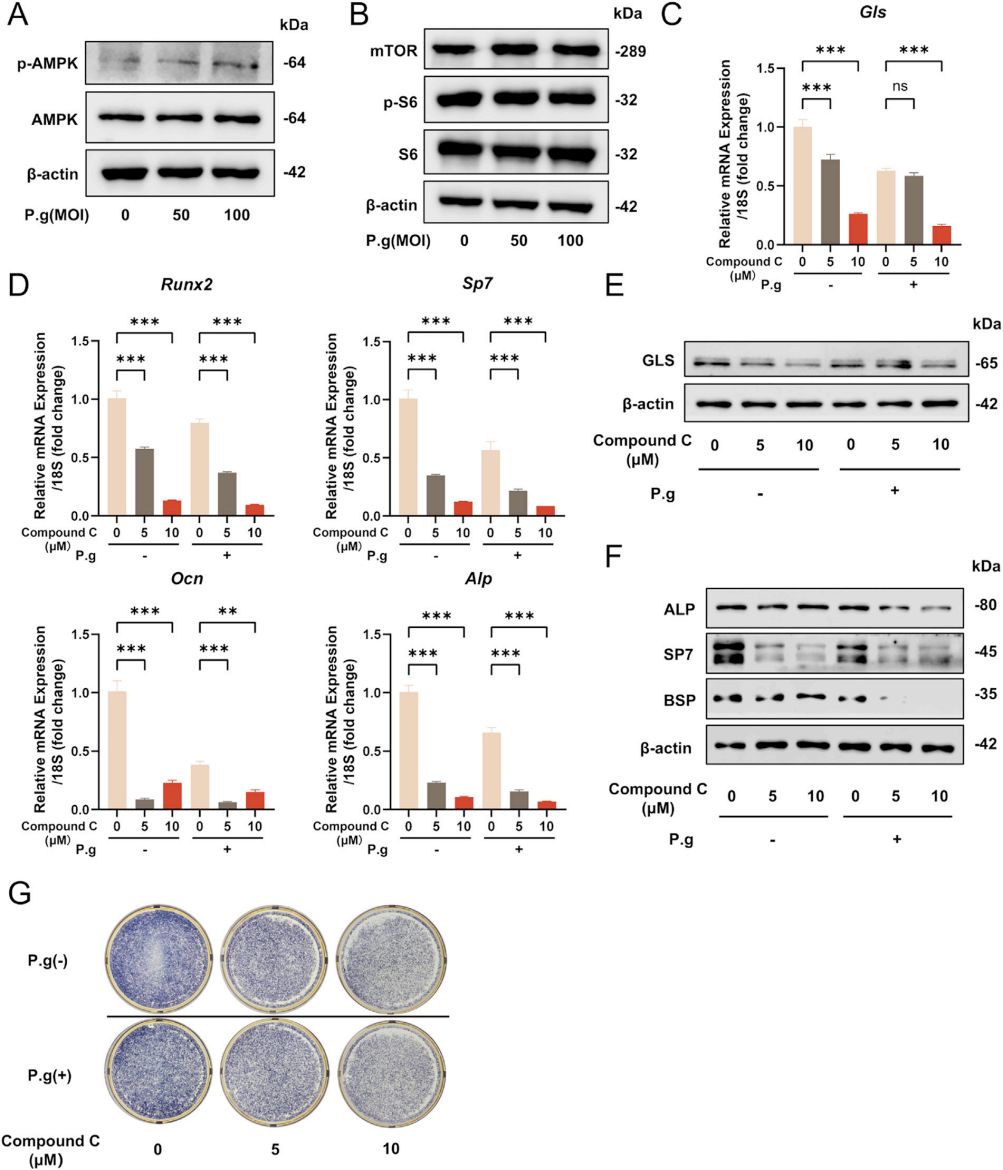
Glutamine metabolism compensates for mineralization by cementoblasts under *P.g* inflammation through the AMPK/GLS pathway. A-B, OCCM-30 cells were treated with different MOIs (MOI = 0, 50, and 100) of *P.g* for 4 days. Protein levels of AMPK, phosphorylated AMPK (p-AMPK) (A) and mTOR, ribosomal protein S6 (S6) and phosphorylated ribosomal protein S6 (p-S6) (B) were detected by Western blot. C, OCCM-30 cells were mineralization induced under different Compound C concentrations (0, 5, and 10 μM) with or without *P.g*, and *Gls* expression level was detected by qPCR. D, *Runx2, Sp7, Ocn* and *Alp* expression levels were detected by qPCR. E, The GLS expression level was detected by Western blot. F, ALP, SP7 and BSP expression levels were detected by Western blot. G, ALP staining was conducted to verify mineralization ability. Data are presented as mean ± SEM. *P < .05, ^⁎⁎^*P* < .01, ^⁎⁎⁎^*P* < .001 and *ns* for not significant. Biological replicates for each experiment, *n* = 3.

AMPK and mTORC1 are two major central kinase complexes that sense intracellular nutrient levels and mutually regulate cell metabolism and growth. Interestingly, the phosphorylation of AMPK increased under *P.g* inflammation (Figure 6A), with no significant change in the mTOR pathway (Figure 6B). To examine the role of the AMPK pathway for mineralization by cementoblasts under *P.g* inflammation, we employed the AMPK inhibitor Compound C to inhibit AMPK activity in cementoblasts. The mRNA levels of *Gls* significantly decreased 2 days after the application of Compound C (0, 5, 10 μM) (Figure 6C), with *Runx2, Sp7, Ocn* and *Alp* expression declining remarkably (Figure 6D). Protein levels of GLS decreased significantly by Compound C in a concentration-dependent manner (Figure 6E) while ALP, SP7, BSP and OCN protein levels also declined remarkably (Figure 6F). The result of ALP staining showed an inhibitory effect of Compound C on mineralization by cementoblasts (Figure 6G). In general, these findings suggest that AMPK has a positive effect on promoting glutamine metabolism and mineralization by cementoblasts.

### Glutamine supplementation restores cementogenesis in mice apical periodontitis

**Fig. 7:**
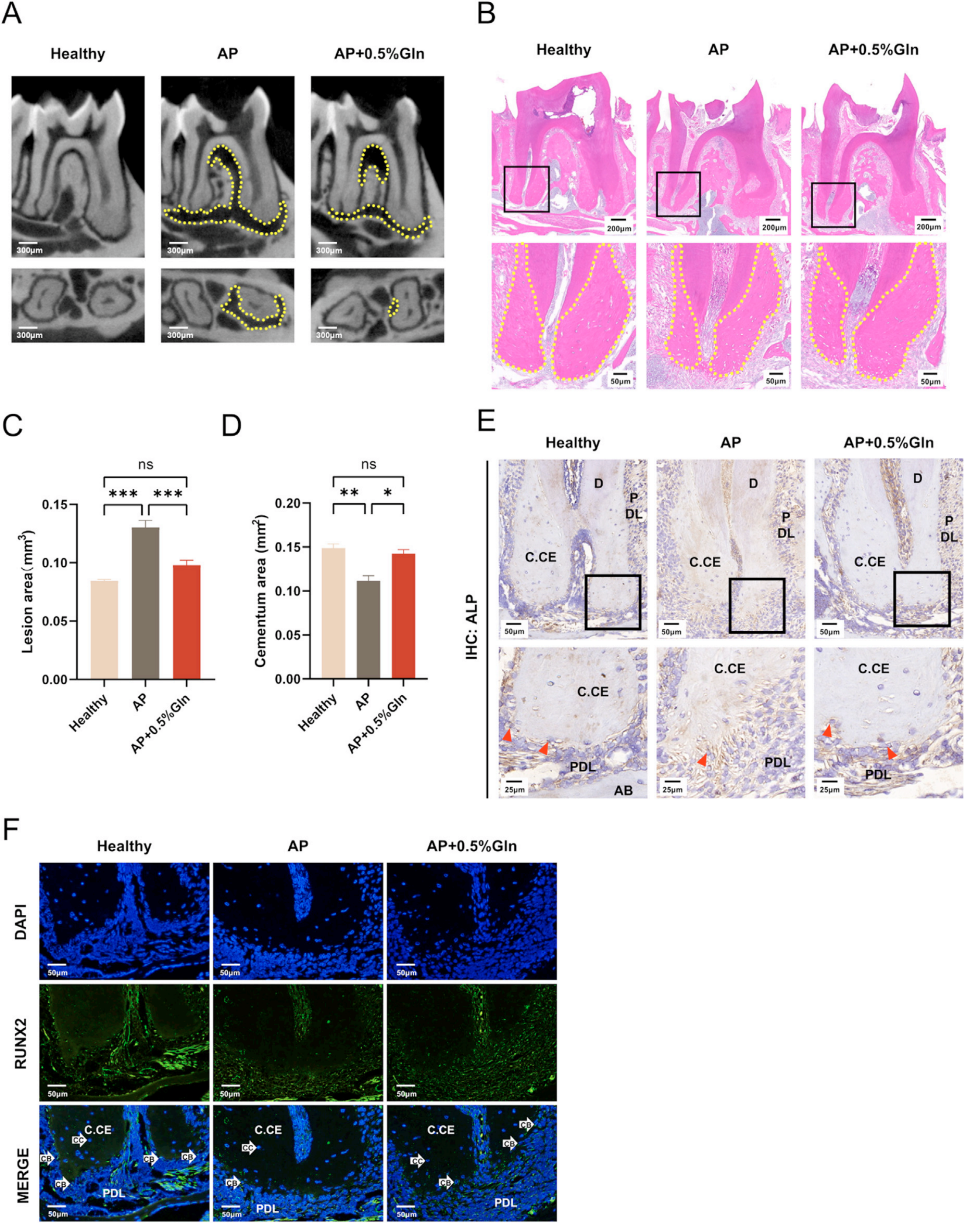
Glutamine supplementation compensates for cementogenesis in mice with apical periodontitis. A, Representative sagittal and transverse micro-CT images of the mandibular first molars in each group. Dashed outlines show the range of bone destruction in the periapical area. B, Representative haematoxylin and eosin (H&E) staining of the mandibular first molars in each group. Dashed outlines indicate cellular cementum. C, Quantification of lesion areas at apical bone destruction in each group derived from quantitative statistics of the micro-CT data in (A). D, Quantification of cementum area in each group derived from quantitative statistics of the H&E staining data in (B). E, Immunohistochemical staining of ALP in the distal root apical region of the mandibular first molars in each group. AP, apical periodontitis; D, dentin; C. CE, cellular cementum; PDL, periodontal ligament; AB, alveolar bone. F, Immunofluorescent staining of RUNX2 in the distal root apical region of the mandibular first molars in each group. CC, cementocyte; CB, cementoblast. Data are presented as mean ± SEM. *P < .05, ^⁎⁎^*P* < .01, ^⁎⁎⁎^*P* < .001 and *ns* for not significant. The number of teeth analysed for CT, *n* = 10; the number of teeth analysed for histological staining, *n* = 3.

Having verified *in vitro* that glutamine supplementation can partially restore the mineralization capacity of cementoblasts under *P.g* inflammation, we constructed apical periodontitis models in mice to explore the potential protective effects of glutamine supplementation *in vivo*. As shown in the representative pictures (Figure 7A), the AP group significantly aggravated the destruction of the periapical root, which was partially relieved by oral glutamine supplementation. The quantitative statistics of the apical lesion area in each group showed the same result as in Figure 7C. In AP mice, H&E staining (Figure 7B) showed a significant reduction of cellular cementum, indicating an inhibitory effect of inflammation on the cementogenesis process, whereas exogenous Gln supplementation alleviated apical cementum destruction, indicating that Gln could promote apical cementum formation. The quantitative statistics of the cementum area in each group showed a significant reduction in the AP mouse in Figure 7D while Gln supplementation alleviated apical cementum destruction. Meanwhile we conducted immunohistochemical staining (Figure 7E), and a lower level of ALP was observed in AP mice, which was partially reversed by extra glutamine supplementation. Furthermore, RUNX2 expression in cementoblasts was detected by immunofluorescent staining (Figure 7F). In AP model mice, RUNX2 fluorescence was significantly decreased in apical cementoblasts, with a partially mitigated decrease in the glutamine-supplemented group. The above results were consistent with the results *in vitro*.

**Fig. 8:**
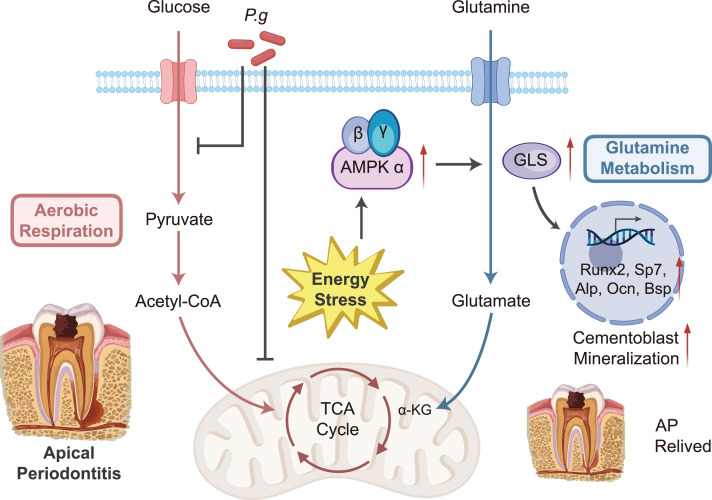
Metabolic changes and molecular mechanisms in cementoblasts under *P.g* infection. *P.g* infection inhibits glycolysis and oxidative phosphorylation in cementoblasts, leading to intracellular energy stress. The AMPK pathway is further activated under this condition, promoting the expression of GLS and enhancing glutamine metabolism, which helps to restore cementoblast mineralization ability.

The above findings proved that in the context of the *P.g* inflammatory environment, activation of the AMPK pathway promotes the expression of GLS, the key enzyme of glutamine metabolism, thereby enhancing glutamine metabolism and partially restoring the mineralization effect of cementoblasts (Figure 8).

## Discussion

This study uncovers a metabolic adaptation mechanism in cementoblasts under *P.g*-induced inflammation, where energy stress triggers AMPK/GLS-mediated glutaminolysis to sustain mineralization. Although *P.g* suppressed glycolysis and oxidative phosphorylation—consistent with its known role in mitochondrial dysfunction^24^^,^^25^—compensatory upregulation of glutamine metabolism emerged as a critical survival strategy. These findings align with skeletal stem cell studies showing glutamine dependency during osteogenesis^32^ but uniquely highlight cementoblast-specific adaptations under microbial challenge.

Glutamine is the most abundant non-essential amino acid in the human body, which plays a very important role in various physiological and pathological situations. Glutamine is transported into the cell and then transformed into glutamate catalysed by GLS, which is decomposed to generate α-KG and further participates in the TCA cycle. M2 macrophages were found secreting glutamate-containing extracellular vesicles and increasing intracellular α-KG production to alleviate osteoporosis.^42^ On the other hand, α-KG is able to activate the mTORC1 signalling pathway, which in turn promotes the M2-type polarisation and proliferation of tumour-associated macrophages (TAMs), indirectly affecting tumour progression.^43^ In our study, untargeted metabolomics revealed a significant reduction of glutamine and glutamate in cementoblasts under *P.g* infection, whereas no significant change in α-KG was observed. So, we conjecture that glutamine metabolism in cementoblasts may not affect the TCA cycle involved by α-KG or energy metabolism. Another possible explanation is that the low-energy environment caused by *P.g* infection activates glutamine metabolism, and the decomposition of glutamine and glutamate is augmented to compensate for the lack of α-KG in the TCA cycle. Given the limitations of snapshot metabolomic analysis, future studies employing isotopic tracing will be necessary to precisely delineate glutamine flux into the TCA cycle under inflammatory conditions.

AMPK, a master regulator of cellular energy homeostasis, is activated in response to intracellular energy depletion to coordinate catabolic processes and restore ATP balance. Emerging evidence highlights the AMPK–PDZD8–GLS1 axis as a pivotal mechanism for metabolic adaptation under energy stress, wherein AMPK activation enhances glutaminolysis to sustain cellular viability.^38^ Our findings align with this paradigm, demonstrating that *P.g*-induced inflammatory stress activates AMPK signalling, which in turn drives GLS upregulation to compensate for impaired glycolysis and oxidative phosphorylation. Notably, while our study establishes a functional link between AMPK activation and GLS expression, the precise mechanism by which AMPK regulates GLS at the transcriptional level remains to be elucidated. Our experiments employed the AMPK inhibitor Compound C to validate the role of the AMPK pathway in GLS expression and mineralization. However, Compound C exhibits certain off-target effects, which may impose limitations on the accuracy of the experimental results. Therefore, AMPK gene knockout or the use of transgenic animal models will be essential in subsequent experiments.

Oral glutamine attenuated apical periodontitis progression in mice, likely by bypassing *P.g*-induced metabolic bottlenecks. This aligns with studies where exogenous glutamine at the tendon–bone interface rescued osteoblast function in osteoporosis models.^44^ Additionally, local glutamine administration has been reported to enhance the antigen-presenting capacity of dendritic cells in tumors, thereby inhibiting tumor growth.^45^ These findings suggest that localized, sustained-release glutamine delivery within the periodontal micro-environment may represent a promising strategy to promote periodontal regeneration. Similar to studies focusing on the differentiation-promoting effects of various drugs on dental pulp stem cells,^46^^,^^47^ our *in vivo* findings provide initial evidence that oral glutamine supplementation can alleviate AP in mice, potentially serving as a preliminary basis for recommending dietary glutamine intake in patients with AP. Overall, as a promising therapeutic modality for the future, our current research findings provide a novel theoretical foundation for the alleviation and treatment of periodontitis and apical periodontitis in the realm of metabolic therapy.

## Conclusion

This study uncovered previously unrecognized metabolic alterations in cementoblasts during inflammatory mineralization. It was found that *P.g* disrupts cementoblast energy metabolism to inhibit mineralization; however, AMPK/GLS-driven glutaminolysis serves as a compensatory mechanism to sustain cell function. Targeting this metabolic pathway may represent a promising therapeutic strategy for periodontal regeneration. Future research should investigate combinatorial approaches that integrate metabolic modulation with antimicrobial therapy to enhance clinical outcomes.

## Conflict of interest

None declared.
